# Long-Term Persistence of Seroprotection by Hepatitis B Vaccination in Healthcare Workers of Southern Italy

**DOI:** 10.5812/hepatmon.6025

**Published:** 2012-09-04

**Authors:** Giuseppe Grosso, Antonio Mistretta, Stefano Marventano, Roberta Ferranti, Luisa Mauro, Rosario Cunsolo, Lidia Proietti, Mariano Malaguarnera

**Affiliations:** 1 G.F. Ingrassia Department, Section of Hygiene and Public Health, University of Catania, Catania, Italy; 2 Rosario Cunsolo, Vittorio Emanuele Hospital of Catania Health Direction, Catania, Italy; 3 Department of Internal Medicine and Systemic Diseases, University of Catania, Catania, Italy; 4 The Great Senescence Research Center, University of Catania, Ospedale Cannizzao, Catania, Italy

**Keywords:** Hepatitis B Virus, Vaccines, Health Personnel, Vaccination

## Abstract

**Background:**

The impact of hepatitis B virus (HBV) vaccination campaigns on HBV epidemiology needs to be evaluated, in order to assess the long-term immunity offered by vaccines against HBV.

**Objectives:**

To evaluate the current status of anti-HBV vaccine coverage among healthcare workers (HCWs) in Southern Italy, and to determine the long-term persistence of antibodies to hepatitis B surface antigens (anti-HBs) in such a cohort of subjects.

**Patients and Methods:**

A longitudinal, retrospective seroepidemiological survey was conducted among 451 HCWs, who were working at or visiting, the Occupational Health Department of a city hospital, in Catania, Italy, between January 1976 and December 2010.

**Results:**

At the 30-year follow-up (mean follow-up 10.15 ± 5.96 years, range 0.74-30), 261 HCWs had detectable anti-HBs titers indicating a persistence of seroprotection of 89.4% (out of 292 anti-HBs positive results, three months after vaccination). An inadequate vaccination schedule was the strongest predictor of antibody loss during follow-up (OR = 8.37 95% CI: 5.41-12.95, P < 0.001). A Kaplan-Maier survival curve revealed that the persistence of anti-HBs 30 years after vaccination, was 92.2% for high responders, while it was only 27.3% for low responders (P = 0.001).

**Conclusions:**

A good level of seroprotection persisted in 57.9% of the subjects after 30 years. Factors related to this immunization status confirmed the importance of vaccinating HCWs early in their careers and ensuring an adequate vaccination schedule. However, with particular reference to the low rate of hepatitis B vaccine coverage among HCWs in Southern Italy, the implementation of a new educational intervention as part of an active vaccination program is needed.

## 1. Background

In Italy, during the 1980s, the hepatitis B virus (HBV) infection was one of the major causes of mortality, leading to about 9 000 deaths per year from HBV-related diseases, such as; chronic active hepatitis, hepatocellular carcinoma, and cirrhosis (1). Hepatitis B infection was a common infectious occupational disease among healthcare workers (HCWs), thus a vaccination campaign was conducted in 1983 to sensitize high-risk groups, such as HCWs, for whom vaccination was strongly suggested (2). However, despite the long-standing existence of recommendations for such high-risk groups (3), hepatitis B vaccinations only reached a small percentage of these populations, who remained susceptible to the virus (4). Indeed, the incidence of hepatitis B in HCWs continued to be higher than in the general population, reflecting poor vaccine coverage (5). This may be attributed to the lack of perceived risk of hepatitis B infection in this job category, or the absence of appropriate healthcare programs targeting vaccination against HBV infection (6). However, a survey conducted in Italy during the 1990s showed an insufficient overall coverage against HBV, with lower rates in the south of the country (7), that was mostly endemic for HBV infection in association with the hepatitis delta virus (8).

In recent years, no difference has been reported in the incidence rate of acute HBV between HCWs and the general population (9), however, this may not necessarily be due to vaccination programs, but to non-specific prophylactic measures such as; more accuracy in hospital procedures, training programs for HCWs, and proper sterilization. Thus, the impact of HBV vaccination campaigns on HBV epidemiology should be evaluated at the present time, with the aim of establishing the long-term immunity offered by vaccines against HBV. Indeed, the antigenic stimulation of the vaccine leads to the production of antibodies to hepatitis B surface antigens (anti-HBs).

## 2. Objectives

The aim of the present study was to evaluate the current status of anti-HBV vaccine acceptance among HCWs in the city of Catania, Southern Italy. Moreover, we aimed to determine the long-term persistence of anti-HBs in such a cohort of subjects.

## 3. Patients and Methods

A longitudinal retrospective seroepidemiological survey was conducted among HCWs of the Vittorio Emanuele Hospital, Catania, Southern Italy, who visited the Occupational Health Department between January 1976 and December 2010, and this process is still ongoing. The study was approved by the ethic committee of the hospital and subjects information were assured of complete anonymity. Data from 956 HCWs were collected. Out of the overall HCW population, only 451 HCWs (mean age 32.8 years, range 25-70 years) had received vaccination and were included in the survey.

### 3.1. Study Population

Each newly employed HCW must undergo a medical examination in the Occupational Health Department prior to signing his or her contract and joining the hospital in their area of their expertise. During such visits, subjects were screened for HBV infection and those having anti-HBs levels less than 10 mIU/mL were referred to be vaccinated.

### 3.2.Vaccination

The vaccine was provided free of charge by the hospital, to all HCWs, but the vaccination itself was not mandatory. The vaccine schedule was delivered by the laboratory staff in the Outpatient Department of the hospital. In most cases, a yeast-derived recombinant HBV vaccine was administered and subjects were advised to take three doses at zero, one, and six months of 1 mL (20 μg/mL) intramuscularly (in the deltoid muscle). We considered ‘vaccinated‘ subjects as having received at least the first vaccination dose, while considering ‘compliant‘ as those who had received the complete schedule. Subjects younger than 12 years old were mandatorily vaccinated during the vaccination campaign introduced by law since 1981, and they did not need to prove that they had been vaccinated from their health records.

### 3.3. Serologic Tests

Blood samples were checked for anti-HBs levels by an enzyme immunoassay (DiaSorin, s.r.l, Saluggia, Italy) during the initial screening of HCWs, and this process was repeated three months after the third dose of anti-HBV vaccine and routinely every two years. Moreover, anti-HBs levels of antibodies were often checked after accidental exposure to blood or needle stick injuries. The serologic status of subjects, who were vaccinated during the vaccination campaign in 1981, was assessed at the first visit after the recruitment, but no data about post-primary immunization titers were available.

### 3.4. Data Collection

Demographic data; age, gender, occupation and working hours, as well as clinical data; BMI, medical history, smoking status, age at primary vaccination, date and concentration (mIU/ml) of the initial anti-HBs antibody measurement, were collected retrospectively by three investigators using a structured form to collect data from the chart of each subject included in the survey. Occupational categories included; physicians (non-surgeon and surgeon), nurses, technicians and laboratory workers. Working hours were divided into day (approximately 4 to 8 consecutive hours, depending if they were full-time or part-time, from 7 am to 9 pm), night (from 9 pm to 7 am), and night and day shift (with varying amounts of night-shift depending on the type of ward) categories. BMI was calculated according to international standards by weight (kg)/height (m^2^) and subjects were considered normal if it was less than or equal to 25, overweight between 25 and 28, and obese if higher than 28. Subjects’ smoking status was classified as; (i) non-smokers, (ii) smoking one to 20 cigarettes per day, and (iii) more than 20 cigarettes per day. Medical history comprised of diseases that may interfere with the immune system (e.g., allergies, asthma, splenectomy, sarcoidosis, autoimmune diseases, chronic viral hepatitis and cancer), and diseases not-known to interfere with the immune system. We considered subjects as seropositive against hepatitis B if their anti-HBs antibody concentration was greater than or equal to 10 mIU/ml, and seronegative if it was less than 10 mIU/ml as recommended by the United States Advisory Committee on Immunization Practices and the World Health Organization (WHO) (10). Antibody titers following primary vaccination were classified into low responders (10–99 mIU/ml) and high responders (≥ 100 mIU/ml). Antibody loss and antibody persistence were defined as testing negative or positive, respectively, for anti-HBs according to the results of the commercial kits.

### 3.5. Statistical Analysis

Categorical variables were presented as frequency of occurrence and percentage and differences between groups were assessed by a chi-square test. Continuous variables were presented as mean and standard deviations, and differences between groups were assessed by a Student’s t-test. The cumulative incidence was calculated using the Kaplan–Meier method. The crude odds ratios (ORs) were used for the association of immunization loss, with the characteristics of subjects evaluated by univariate analysis. Adjusted ORs and their 95% confidence intervals were calculated by stepwise logistic regression analysis to identify independent predictors of becoming seronegative. Only variable results associated at univariate analysis were entered into the logistic model. P ≤ 0.05 was considered significant. Analyses were carried out using the Statistical Package for the Social Sciences version 17.0 (SPSS, Chicago, IL, USA). 

## 4. Results

**Figure 1:**
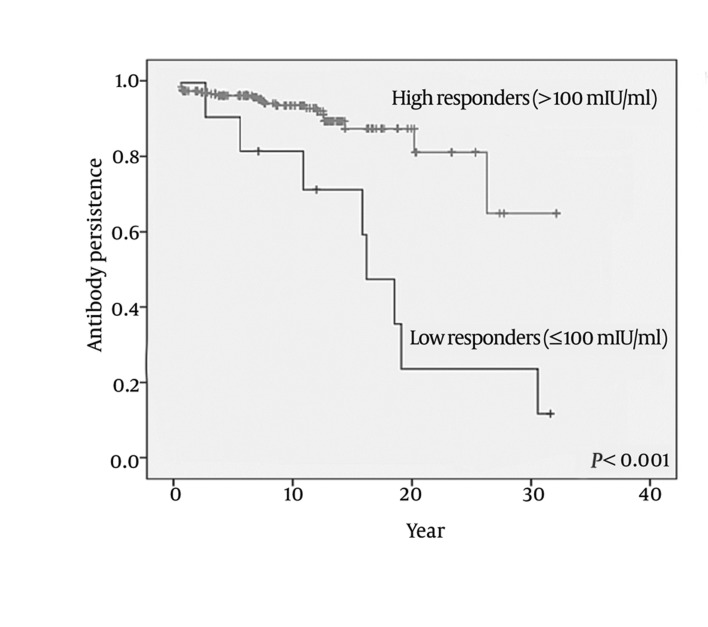
Anti-HBsAg Persistence According to Post-Vaccination Anti-HBsAg Titers

Sociodemographic and medical characteristics of the 451 HCWs included in the study are listed in Table 1. Fully compliant subjects with a completed vaccination schedule consisted of 157 (34.8%) HCWs. Three months following vaccination, the overall anti-HBs prevalence in vaccinated HCWs was 64.7% (292 subjects). Vaccination at a young age, either at birth or before the age of 12, as well as having received an inadequate schedule of vaccination, were significantly associated with being seronegative when tested (P < 0.0001). Among the 181 subjects vaccinated at job commencement or during their working life, the response after primary immunization was 52.1% (158 subjects). At the 30-year follow-up (mean follow-up 10.15 ± 5.96 years, range 0.74-30), 261 HCWs (57.9% out of the overall 451 vaccinated HCWs) had detectable anti-HBs titers, indicating a persistence of seroprotection of 89.4% (out of the 292 anti-HBs positive HCWs three-months after vaccination). Characteristics significantly associated with becoming seronegative are listed in Table 2. Younger and overweight HCWs with 10-20 years in their occupation were less likely to lose seroprotection against HBV (OR = 0.47, 95% CI: 0.3-0.72, P = 0.01; OR = 0.7, 95% CI: 0.51-0.95, P = 0.022 and OR = 0.65, 95% CI: 0.43-0.98, P = 0.41, respectively), compared with older, normal weight HCWs employed for less than 10 years. By contrast, an inadequate vaccination schedule was found to be most strongly associated with antibody loss during follow-up, even after adjusting for other significant covariates (OR = 8.37, 95% CI: 5.41-12.95, P < 0.001). Results of anti-HBs titer levels detected in subjects vaccinated at job commencement or during their working life were similar to those for all vaccinated HCWs (Table 2). At the 30-year follow-up, the odds for antibody loss were higher in subjects that had received an inadequate schedule (OR = 16.97, 95% CI: 7.12-40.41, P < 0.001). Kaplan-Maier survival curve of time from primary vaccination to an anti-HBs antibody measurement outcome less than 10 mIU/ml, revealed that low post-immunization titers (10–99 mIU/ml) was a predictor of antibody loss during the follow-up period. The persistence of anti-HBs 30 years after vaccination was 92.2% for high responders, while it was only 27.3% for low responders (P < 0.001) (Figure 1). When analyzing risk factors associated with becoming seronegative against hepatitis B, being a low-responder after primary immunization was a predictor of antibody loss after the follow-up period (OR = 6.18, 95% CI: 2.48-17.01, P < 0.001), but after adjusting for the vaccination schedule, only the last factor remained significant (OR = 11.76, 95% CI: 2.99-46.2, P < 0.001).

**Table 1:** Demographic, Occupational and Clinical Characteristics of the Study Population by Serologic Status

	**Seropositive, No. (%) (n = 292)**	**Seronegative, No. (%) (n = 159)**	**Total, No. (%) (n = 451)**	***P *value**
**Gender**				0.549
Male	153 (52.4)	88 (55.3)	241 (53.4)	
Female	139 (47.6)	71 (44.7)	210 (46.6)	
**Age group, y**				< 0.0001
25-35	204 (69.9)	139 (87.4)	343 (76.1)	
36-45	73 (25)	14 (8.8)	87 (19.3)	
> 45	15 (5.1)	6 (3.8)	21 (4.7)	
**BMI, kg/m^2^**				0.277
> 25	120 (41.1)	54 (34)	174 (38.6)	
> 30	13 (4.5)	6 (3.8)	19 (4.2)	
**Medical history**				0.131
Group A ^a^	187 (64)	113 (71.1)	300 (66.5)	
Group B ^b^	105 (36)	46 (28.9)	151 (33.5)	
**Smoking status**				0.745
Non smoker	163 (55.8)	89 (56)	252 (55.9)	
1-20 cigarettes per day	101 (34.6)	58 (36.5)	159 (35.3)	
> 20 cigarettes per day	28 (9.6)	12 (7.5)	40 (8.9)	
**Occupational category**				0.794
Physician	46 (18.6)	21 (17.5)	67 (18.3)	
Nurse	201 (81.4)	99 (82.5)	300 (81.7)	
**Hospital department**				0.782
Medical department	117 (45.5)	53 (42.1)	170 (44.4)	
Surgical department	130 (50.6)	67 (53.2)	197 (51.4)	
Laboratory department	10 (3.9)	6 (4.8)	16 4.2)	
**Years in occupation**				0.1
> 10	233 (79.8)	144 (90.6)	377 (83.6)	
20-Oct	55 (18.8)	13 (8.2)	68 (15.1)	
> 20	4 (1.4)	2 (1.3)	6 (1.3)	
**Working schedule**				0.679
Day shift	193 (66.1)	103 (64.8)	296 (65.6)	
Night shift	24 (8.2)	17 (10.7)	41 (9.1)	
Rotating day/night shift	75 (25.7)	39 (24.5)	114 (25.3)	
**Vaccine status**				< 0.0001
Vaccinated = 12 years	140 (47.9)	130 (81.8)	270 (59.9)	
Vaccinated > 12 years	152 (52.1)	29 (18.2)	181 (40.1)	
**Vaccination schedule**				< 0.0001
Adequate	267 (91.4)	27 (17)	294 (65.2)	
Inadequate	25 (8.6)	132 (83)	157 (34.8)	

^a^Diseases not known to interfere with the immune system

^b^Diseases that could interfere with the immune system

**Table 2:** Factors Associated With Becoming Seronegative Against Hepatitis B by Vaccination Status

	** Overall Vaccinated (n = 451)**				**Vaccinated > 12 years (n = 181)**			
	**Unadjusted OR (95 % CI), No. (%)**	**P value**	**Adjusted OR (95 % CI), No. (%)**	***P *value**	**Unadjusted OR (95 % CI) , No. (%)**	***P* value**	**Adjusted OR (95 % CI), No. (%)**	***P* value**
**Gender**							-	
Male	1	-	-	-	1	-	-	-
Female	1.01 (0.76-1.35)	0.918	-	-	1.02 (0.56-1.84)	0.952	-	-
**Age group, y**								
25-35	1	-	1	-	1	-	-	-
36-45	0.47 (0.3-0.72)	0.001	0.68 (0.37-1.26)	0.228	1.29 (0.6-2.75)	0.507	-	-
> 45	0.54 (0.3-0.97)	0.043	0.49 (0.18-1.32)	0.16	1.46 (0.6-3.58)	0.401	-	-
**BMI, kg/m^2^**								
< 25	1	-	1	-	1	-	-	-
25-28	0.7 (0.51-0.95)	0.022	0.71 (0.5-1)	0.054	0.67 (0.37-1.22)	0.196	-	-
> 28	0.6 (0.28-1.3)	0.191	0.61 (0.22-1.67)	0.335	0 (0)	0.978	-	-
**Medical history**								
Group A ^a^	1	-	-	-	1	-	-	-
Group B ^b^	0.86 (0.63-1.17)	0.328	-	-	1.12 (0.61-1.2)	0.729	-	-
**Smoking status**								
Non smoker	1		-	-	1	-	-	-
1-20 cigarettes/day	0.92 (0.67-1.25)	0.601	-	-	0.58 (0.3-1.12)	0.109	-	-
>20 cigarettes/day	1.06 (0.63-1.77)	0.819	-	-	0.69 (0.16-2.93)	0.62	-	-
**Occupational category**								
Physician	1	-	-	-	1	-	-	-
Nurse	1.06 (0.7-1.6)	0.78	-	-	1.38 (0.61-3.12)	0.431	-	-
**Hospital department**								
Medical	1	-	1	-	1	-	-	-
Surgical	1.53 (1.09-2.12)	0.012	1.36 (0.97-1.91)	0.072	1.61 (0.88-2.95)	0.117	-	-
Laboratory	1 (0.46-2.54)	0.846	0.45 (0.2-1.08)	0.076	0 (0)	1	-	-
**Years in occupation**								
< 10	1	-	1	-	1	-	-	-
10-20	0.65 (0.43-0.98)	0.041	0.96 (0.47-1.99)	0.93	1.62 (0.88-3)	0.119	-	-
> 20	0.34 (0.1-1.08)	0.068	0.51 (0.12-2.21)	0.373	0.7 (0.19-2.57)	0.597	-	-
**Vaccination schedule**								
Adequate	1	-	1	-	1	-	1	-
Inadequate	7.5 (5.14-10.96)	< 0.001	8.37 (5.41-12.95)	< 0.001	16.97 (7.12-40.41)	< 0.001	11.76 (2.99-46.2)	< 0.001
**Immunization**								
High responder	-	-	-	-	1	-	1	-
Low responder	-	-	-	-	6.18 (2.48-17.01)	< 0.001	1.14 (0.31-4.1)	0.841

^a^Diseases not known to interfere with the immune system

^b^Diseases that could interfere with the immune system

## 5. Discussion

This longitudinal, retrospective seroepidemiological survey aimed to collect and analyze important information on vaccination against HBV in a hospital environment, including compliance rates and serological status of those vaccinated. Based on our knowledge, this is the first study to assess compliance rates of vaccination and markers kinetics over a follow-up period of 30 years. Moreover, this is the first report that underlines the situation of immunization status against HBV, due to poor compliance rates to the vaccine, among HCWs in Sicily. Indeed, according to our study, the current overall vaccine coverage among HCWs in Catania was only 61.2% (451 of 956). Several other studies have assessed lower vaccination rates among HCWs in Southern Italy than in the northern regions, but in our opinion these works underestimated this trend. In fact, in Southern Italy overall vaccine coverage has been documented to have increased from 44.3% in a survey conducted in 1996 (7), to 77.7% in another survey conducted in 2006 (11), however, our findings are much lower than these results. Moreover, taking into account the introduction of the compulsory-by-law HBV vaccination in Italy for all newborns in 1981 and 12-year-old children in 1991, as well as HCWs vaccinated only after a percutaneous exposure incident, we can conclude that compliance rates to the vaccination against HBV among HCWs in Southern Italy at the start of their employment, considered as a positive attitude towards the vaccination program, were even lower. Accordingly, compared with other countries, compliance rates in our study are similar to those reported in the USA (54%) (12), Spain (47.1%) (13), Australia (55.8%) (14), and Nigeria (53.8%) (15), but lower than those reported in the UK (80%) (16), and Brazil (80.7%) (17). According to the job category, physicians were shown to be compliant, as well as nurses. Compliance rates among physicians assessed in our study were similar to those reported in; Australia (14), the UK (16), and Brazil (17), ranging between 69% and 98.6%, much higher than those reported elsewhere, which range from 30% to 40.3% (12, 15). However, an indifferent attitude in regard to hepatitis B vaccine among physicians is also well recognized (18-20), resulting in doctors being relatively overlooked, compared with other professional groups, with regard to both education and vaccination (21, 22).

Among the 181 HCWs vaccinated at job commencement or after, we analyzed antibody kinetics to assess whether subjects were still immunized during a follow-up period of 30 years, finding that the cumulative percentage of seroprotection was 52.1%. The persistence of anti-HBs antibodies is well documented and our study seems to confirm existing findings in the literature (23, 24). However, how long immunity might be expected to last after vaccination and its role in immunization is still a controversial issue. Indeed, no scientific evidence supports the assumption that immunity depends entirely on anti-HBs antibodies. Thus, according to the WHO, individuals with anti-HBs levels which dropped below 10 mIU/ml are supposed to remain immunized against HBV, due to the stronger role in the anamnestic response of cellular immunity. On the other hand, it has been documented that subjects with post-vaccination anti-HBs levels lower than 50 mIU/ml may contract a HBV infection, experience hepatitis B surface antigen reactivity (25), or develop the disease. Thus, in European countries the threshold which considers that a subject is immunized, is recommended to be at least 100 mIU/ml (26). However, we recorded no cases of hepatitis B in non-responder subjects or in those with anti-HBs levels lower than 10 mIU/ml or 50 mIU/ml after vaccination.

The issues concerning anti-HBs levels and the persistence of seroprotection are important in determining the potential need for booster doses. Although current data on children leads to the assumption that no booster dose is needed before 10 years of age (27), the need for booster doses to maintain HBV seroprotection is still debated in adults, given that results from previous surveys conducted in adult HCWs are in contrast to international guidelines (24, 28, 29). However, subjects vaccinated at 12 years or earlier, and those who did not receive the booster doses at job recruitment, were seronegative at the first visit. Seropositivity status was influenced by the age of subjects at vaccination. Indeed, a younger age at primary vaccination was predictive of seropositivity, confirming the recommendations of the WHO (30). This finding is supported by other studies conducted on HCWs (28, 31, 32). We checked if demographic and clinical variables had an effect on serological status. Being overweight (33, 34, 35, 36), male (37), having a chronic disease (38, 39), and smoking (33), have previously been documented as predictive of seronegative status after vaccination. In our study, univariate analysis only confirmed that being overweight was a predictor of failed response to primary vaccination, while being a low-responder had a higher risk of becoming seronegative during the follow-up period. However, none of these variables remained significant in the multivariate analysis. Among the occupational data, we decided to include the type of occupation and working hours, thinking that such variables could influence immunization status over time, due to the increased risk of HBV infection among different groups, but no significant predictive variable was found. Findings of retrospective seroepidemiological surveys such as ours must be read in light of several limitations. Missing data due to the retrospective nature of the work should be taken in to account. We have also included only those subjects who are still working at the hospital, and whose medical data was still available (ie, excluding fired, deceased, and retired subjects), which may lead to a potential bias. However, detailed information about the time of vaccination as well as serologic data performed soon after vaccination, has been documented to reliably determine whether a seronegative subject was primary (anti-HBs < 10 mIU/ml since vaccination), or secondary (anti-HBs < 10 mIU/ml after a certain time following the vaccination), non-responders to anti-HBV vaccine, and if variables supposed to be associated to seronegativity were related to becoming or staying seronegative. Conversely, a limitation of this study in regard to the role of such variables occurred, because they were collected at the time of recording the HCW’s chart, thus we cannot assure the status of the subject at the time of vaccination and we can only hypothesize that characteristics collected at the start of their work and joining the hospital staff, remained stable as far as possible over time.

In conclusion, the implementation of universal precautions such as; safety procedures in hospitals pre-dating the availability of anti-HBV vaccine, were associated with decreased high-risk exposures and the decreased incidence of HBV (40, 41), leading to a certain degree of controversy when attributing the lower rates solely to HBV vaccination programs (42). On the other hand, several studies have shown that a vaccination program in healthcare workers against HBV was cost-effective, decreased the anxiety of an employee after needle stick and sharp injuries, and prevented the transmission of HBV after exposure in the majority of cases (3, 43, 44). Thus, according to the results of this study, a good level of seroprotection persisted in 57.9% of subjects after 30 years, and factors related to immunization status confirmed the importance of vaccinating HCWs early in their career and assuring an adequate vaccination schedule. However, with particular reference to the low rate of hepatitis B vaccine coverage among HCWs in Southern Italy, implementation of new educational interventions as part of an active vaccination program is still required.
